# Asiaticoside promotes intestinal epithelial proliferation and barrier function in ischemia/reperfusion injury by activating FoxM1

**DOI:** 10.3389/fphar.2026.1762507

**Published:** 2026-02-18

**Authors:** Chenglin Zhao, Qingyu Du, Yuhang Wu, Xiangwen Zhang, Guo Zu

**Affiliations:** 1 Department of Gastroenterology Surgery, Central Hospital of Dalian University of Technology (Dalian Municipal Central Hospital), Dalian, China; 2 Department of Graduate School, Dalian Medical University, Dalian, China

**Keywords:** asiaticoside, barrier function, FoxM1, intestinal ischemia/reperfusion injury, proliferation

## Abstract

The most important component of intestinal ischemia/reperfusion (II/R) injury is damage to the intestinal mucosal barrier. In II/R injury, damage and restoration occur simultaneously. To develop a treatment for II/R injury, further knowledge about the restoration of intestinal barrier function is needed. Whether asiaticoside (AS) has positive effects on barrier function following II/R injury is unclear, although multiple studies have reported that AS enhances intestinal recovery after injury. In our study, we discovered that AS can reduce the intestinal Chiu score after II/R injury (*P* < 0.05), increase intestinal barrier-associated protein expression (*P* < 0.05) and increase PCNA and Ki-67 expression after II/R injury (*P* < 0.05). Furthermore, following II/R injury, AS primarily activates FoxM1 expression, which promotes cell proliferation and enhances barrier function. TST (a FoxM1 inhibitor) administration significantly reversed the upregulation of FoxM1, as well as the intestinal epithelial proliferation and barrier function induced by AS pretreatment in rats after II/R injury. Therefore, our results reveal that AS promotes cell proliferation and barrier function by activating FoxM1 expression. Our findings may provide a new potential therapeutic approach for treating II/R injury with AS.

## 1 Introduction

Intestinal ischemia/reperfusion (II/R) injury is a common pathophysiological process associated with significant morbidity and mortality in the clinic caused by blood flow recovery following ischemic injury (Hu et al., 2022; Tan et al., 2025). Intestinal ischemia leads to increased microvascular permeability and intestinal epithelial cell and barrier function injury, and reperfusion results in severe local and systemic inflammation and oxidative stress responses, which can induce multiple organ dysfunction syndrome (Zhang et al., 2022; Szabó et al., 2006). Intestinal epithelial cell damage and mucosal barrier failure are key features of II/R injury. In addition, following ischemia/reperfusion (I/R) injury, the gut depends on therapeutic restoration of intestinal barrier function, which necessitates epithelial proliferation, differentiation, and migration.

The pathogenesis of intestinal epithelial cell proliferation following II/R injury is complex and related to many factors and signaling pathways (Chen et al., 2013). Increased epithelial cell proliferation can contribute to enhanced intestinal mucosal barrier function. Multiple signaling pathways are associated with intestinal epithelial cell proliferation and the restoration of mucosal barrier function following II/R injury (Li et al., 2023; Liu et al., 2018). The Forkhead box protein M1 (FoxM1) is a transcription factor and pivotal regulator of cell proliferation. FoxM1 is highly expressed in many organs following injury, including the intestines (Wang et al., 2025), and FoxM1 plays an important role in colon tumorigenesis (Han et al., 2020). Growing evidence have indicated that FoxM1 can effectively attenuate I/R injury in the brain, heart and kidney (Matei et al., 2018; Song et al., 2025; Sinha et al., 2020). We have also reported that FoxM1 promotes intestinal epithelial cell proliferation and barrier function recovery following II/R injury by activating Nurr1 (Zu et al., 2019). Therefore, FoxM1 is considered a potential treatment target for II/R injury.

**FIGURE 1:**
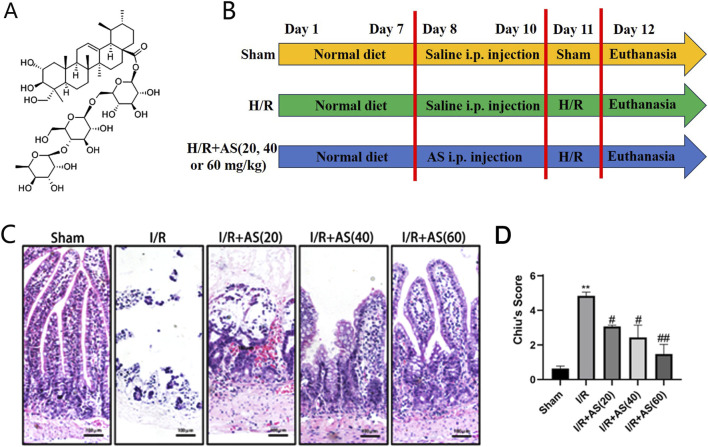
Effects of AS on the microscopic structure of intestinal tissues after I/R injury. **(A)** Chemical structure of AS. **(B)** Experimental protocol. **(C)** Representative micrographs of intestinal tissue (bar = 100 μm). **(D)** Chiu scores of the different groups (n = 5). **P < 0.01 versus sham, ^#^ *P* < 0.05 versus I/R, ^##^ *P* < 0.01 versus I/R.

*Centella asiatica*, a plant in the Apiaceae family, is commonly found as a dry, whole plant or as a rooted whole plant and is also known as Thunder God Root in China. Asiaticoside (AS) belongs to the pentacyclic triterpenoid class of compounds (Figure 1A), which exhibit various pharmacological effects, such as anti-ulcer, wound healing, anti-tumor, anti-inflammatory, and immune regulation (Liu et al., 2024). Our previous study investigated the biological activity of AS in lung injury induced by II/R and revealed that AS can protect against II/R-induced lung injury by activating FoxM1 (Zheng et al., 2024). AS can treat scleroderma and skin wounds and burns by promoting cell proliferation (Kimura et al., 2008). However, whether AS can effectively promote intestinal epithelial cell proliferation and recovery of mucosal barrier function after II/R injury remains unknown. Our study aimed to clarify the effect of AS on intestinal epithelial cell proliferation and recovery of mucosal barrier function after II/R injury.

## 2 Materials and methods

### 2.1 Animals

Healthy, specific pathogen-free (SPF) SD rats (6–8-week-old males, 180–200 g in weight) were purchased from the SPF Experimental Animal Center of Dalian Medical University. The rats were divided randomly into five groups with ten rats in each group as follows: (1) Sham group; (2) II/R group; (3) II/R + AS 20 mg/kg group; (4) II/R + AS 40 mg/kg group; and (5) II/R + AS 60 mg/kg group. Each rat was administered 1 mL 0.9% normal saline containing different concentrations of AS (purity >98%, Sigma Aldrich, USA) once a day via i.p. injection for 3 days.

The II/R injury model was established as described in our previous study (Zheng et al., 2024). Briefly, the rats were anesthetized with an intraperitoneal injection of 2% pentobarbital sodium at a dose of 0.2 mL/100 g bodyweight. After anesthesia, we occluded the superior mesenteric artery with a microvascular clamp for 60 min and then removed the clamp for reperfusion for 6 h. After reperfusion, the rats were euthanized by blood extracted from abdominal aorta following anesthesia as above, and the ileal tissue was removed and then fixed with 4% paraformaldehyde or quickly frozen using liquid nitrogen (Figure 1B).

For our investigation, fifty rats were randomly divided into five groups (n = 10 per group): (1) Sham group; (2) II/R group; (3) II/R + AS (60) group; (4) II/R + AS + thiostrepton (TST, FoxM1 inhibitor; Sigma-Aldrich, USA) group; and (5) II/R + TST group. Fifty mg/kg TST was given once a day via i.p. injection for 2 days prior to II/R surgery.

### 2.2 Histopathological examination

Following paraffin embedding, the separated ileal segment was fixed in paraformaldehyde before being subjected to hematoxylin–eosin (HE) staining. Similar to a prior study, the pathological alterations of the ileum were examined under a microscope and evaluated using Chiu’s scale.

### 2.3 Immunohistochemistry (IHC)

IHC was carried out in accordance with the protocol reported in a prior study (Li et al., 2021). The intestinal tissue slices were incubated with anti-Ki-67 antibody (1:200, Abcam, UK) overnight. After being cleaned, the sections were treated with a secondary antibody. The manufacturer’s instructions were followed for using a streptavidin-biotin-peroxidase kit (ZSGB-BIO, Beijing, China).

### 2.4 Western blotting

Total protein was extracted from intestinal tissues and IEC-6 cells, and the BCA technique was used to quantify the protein samples. After being separated using 12% SDS–PAGE, the protein samples were transferred to PVDF membranes. Following membrane blocking, the membranes were incubated with primary antibodies against FoxM1 and β-actin (1:1000). After the PVDF membranes were washed, a secondary antibody labeled with HRP (1:1000 dilution) was added. The membranes were then analyzed using an ECL detection apparatus. The corresponding sample protein expression of β-actin was used to standardize the protein quantification.

### 2.5 Intestinal permeability assay

An earlier description of intestinal permeability testing was provided. Intestinal barrier permeability was measured using fluorescein isothiocyanate (FITC)-dextran fluorescence intensity. Immediately preceding the ligation of the superior mesenteric artery or collateral vasculature, 200 μL of PBS containing 25 mg/mL FD4 was administered orally. A 100 μL blood sample was drawn following the reperfusion period. A fluorescence spectrophotometer was used to measure the fluorescence intensity of FD4 in plasma.

### 2.6 Immunofluorescence

After being fixed and permeabilized, IEC-6 cells were treated with an anti-PCNA antibody. After the cells were washed, they were incubated with 4′,6-diamidino-2-phenylindole (DAPI) and secondary antibodies. The average number of PCNA-positive enterocytes per 100 enterocytes was calculated and used to assess enterocyte proliferation.

### 2.7 Cell viability assessment

IEC-6 cells (1 × 10^5^/well) were grown and plated into 96-well plates. Following IEC-6 cell treatment, cell viability was assessed using a cell counting kit-8 (CCK-8) assay in accordance with the manufacturer’s instructions (Li et al., 2021). Briefly, 10 μL of CCK-8 was added, and the mixture was incubated for 2 hours. The optical density (OD) was measured. The OD value is a measure of cell viability.

### 2.8 RT–qPCR

Total RNA was isolated from rat intestinal tissues using TRIzol reagent (Takara, Japan), and a Revert Aid First Strand cDNA Synthesis Kit (Thermo Fisher Scientific, USA) was used to generate the first strand of cDNA. Fluorescence quantitative PCR was performed using SYBR Green PCR Master Mix (Monad, China) and a Light Cycler R® 480 (Roche, Switzerland). The following primer sequences were used: FoxM1 F: 5′-CAA​GGT​AAA​AGC​CAC​GTC​TAA​GC-3′, R: 5′-GGAGCAG CAGGTGACTAATGG-3′ and β-actin F: 5′-CTG​GAG​AAG​AGC​TAT​GAG​CTG-3′, R: 5′-AATCT CCTTCTGAT CCTGTC-3'. β-actin served as the internal control.

### 2.9 Statistical analysis

We used the mean ± SD to evaluate the experimental outcomes. One-way ANOVA and a subsequent Tukey test were used to compare the results between three or more groups. *P* < 0.05 was considered to indicate statistical significance.

## 3 Results

### 3.1 Reduced intestinal morphological damage caused by II/R

HE staining revealed that the intestinal mucosal epithelium in the Sham group exhibited distinct epithelial cells and that their shape and texture were intact. Compared with those in the sham group, the intestinal mucosal epithelium in the II/R damage group exhibited significant morphological changes and lamina propria breakdown, and the Chiu score in the II/R group was greater than that in the Sham group. Furthermore, pretreatment with AS (20, 40, or 60 mg/kg) significantly increased the Chiu score, which subsequently decreased in a dose-dependent manner (Figures 1C,D).

### 3.2 AS promoted intestinal mucosal cell proliferation and ameliorated intestinal barrier function injury in rats after II/R injury

**FIGURE 2:**
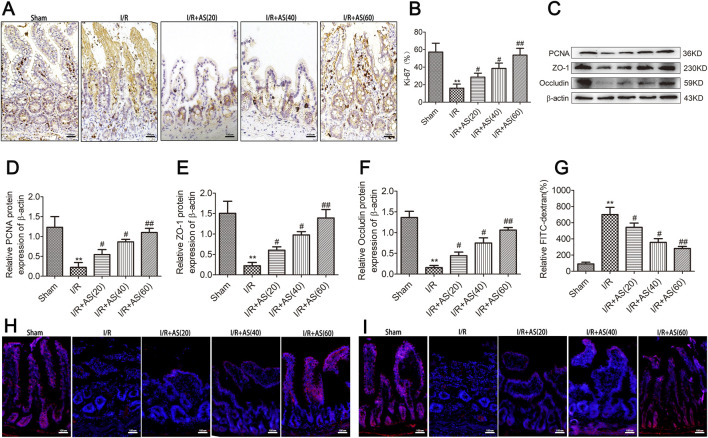
AS improves intestinal mucosal proliferation and barrier function after II/R injury. **(A,B)** IHC staining for Ki-67 expression in intestinal tissues from different groups (bar = 100 μm) (n = 5). **(C–F)** Representative Western blot showing PCNA, ZO-1 and occludin protein expression (n = 3). **(G)** Relative levels of FITC-dextran in intestinal tissues from different groups. **(H)** Immunofluorescence staining for occludin expression in intestinal tissue in the different groups (bar = 100 μm). **(I)** Immunofluorescence staining for ZO-1 expression in intestinal tissue in different groups (bar = 100 μm). **P < 0.01 versus sham, ^#^ *P* < 0.05 versus I/R, ^##^ *P* < 0.01 versus I/R.

Intestinal mucosal cell proliferation was assessed by evaluating the expression of Ki-67 and PCNA using immunohistochemistry and Western blot analyses. The degree of intestinal epithelial barrier damage was assessed by examining tight junction protein expression and intestinal permeability. Our results revealed that the expression of Ki-67 (Figures 2A,B), PCNA and tight junction proteins was significantly downregulated in the group with II/R injury (Figures 2C–F), and AS pretreatment reversed the dysregulation of intestinal epithelial proliferation and tight junction protein expression. Furthermore, intestinal permeability assays revealed that the intestinal epithelial barrier dysfunction induced by II/R could be reversed by AS pretreatment in a dose-dependent manner (Figure 2G). Immunofluorescence staining for tight junction proteins showed the same results of Western blot (Figures 2H,I). Our results indicate that AS pretreatment could alleviate II/R-induced intestinal epithelial cell proliferation and barrier function injury.

### 3.3 AS promoted cell proliferation and ameliorated barrier function in IEC-6 cells after hypoxia/reoxygenation (H/R) injury

**FIGURE 3:**
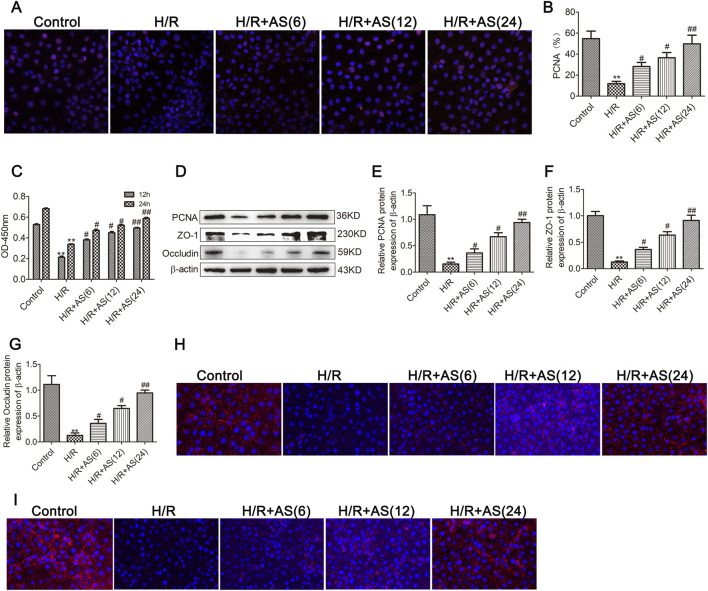
AS promotes IEC-6 cell proliferation and barrier function after H/R injury. **(A,B)** Immunofluorescence staining for PCNA expression in IEC-6 cells for proliferation analysis (n = 6). **(C)** CCK-8 assay to examine cell proliferation at the indicated time points (n = 6). **(D–G)** Representative Western blot showing PCNA, ZO-1 and occludin protein expression (n = 3). **(H)** Immunofluorescence staining for occludin expression in the different groups. **(I)** Immunofluorescence staining for ZO-1 expression in different groups. **P < 0.01 versus control, ^#^ *P* < 0.05 versus H/R, ^##^ *P* < 0.01 versus H/R.

To further validate the effects of AS on intestinal mucosal cell proliferation and barrier function following II/R injury, we examined the effects of AS on cell proliferation and barrier function in IEC-6 cells after H/R injury. Immunofluorescence and CCK-8 assays revealed that the expression of PCNA in the H/R group was lower than that in the control group, and the expression of PCNA in the AS pretreatment group was greater than that in the H/R group (Figures 3A–C). Western blot analysis revealed that the expression of PCNA and tight junction proteins was significantly downregulated in IEC-6 cells after H/R injury, and AS pretreatment reversed the dysregulation of intestinal epithelial cell proliferation and tight junction protein expression (Figures 3D–G); moreover, the immunofluorescence results for occludin and ZO-1 expression were the same as the Western blot results (Figures 3H,I). These results are consistent with those obtained in rats subjected to II/R injury. Therefore, the results indicate that AS pretreatment could alleviate II/R-induced intestinal epithelial cell proliferation and barrier function injury.

### 3.4 AS upregulated the expression of FoxM1 in II/R-injured intestinal tissue

**FIGURE 4:**
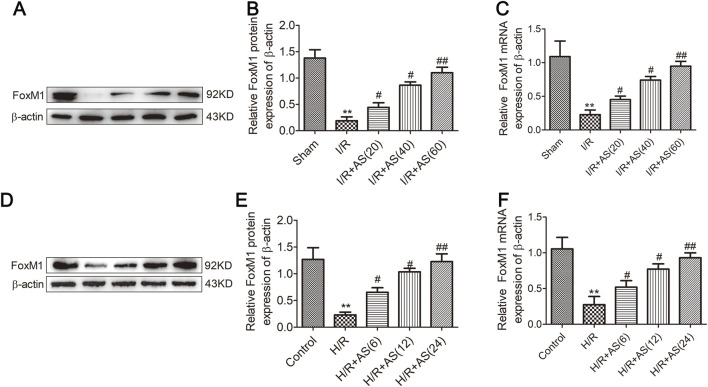
Protein and mRNA expression of FoxM1 in intestinal tissues from different groups. **(A,B)** Protein expression of FoxM1 in intestinal tissues (n = 3). **(C)** mRNA expression of FoxM1 in intestinal tissues (n = 3). **(D,E)** Protein expression of FoxM1 in IEC-6 cells (n = 3). **(F)** mRNA expression of FoxM1 in IEC-6 cells (n = 3). **P < 0.01 versus sham or control, ^#^ *P* < 0.05 versus I/R or H/R, ^##^ *P* < 0.01 versus I/R or H/R.

Our previous studies revealed that FoxM1 plays a crucial protective role in the recovery of intestinal mucosal cell proliferation and barrier dysfunction induced by II/R (Wang et al., 2025). FoxM1 promotes the recovery of intestinal mucosal epithelial cell proliferation and barrier function damage induced by II/R by activating Nurr1 (Zu et al., 2019). AS plays a crucial role in mitigating II/R injury-induced lung injury by promoting FoxM1 expression (Zheng et al., 2024). Therefore, we hypothesized that AS promotes the recovery of intestinal mucosal epithelial cell proliferation and barrier function after II/R injury via FoxM1. To this end, we examined FoxM1 protein and mRNA expression in rats subjected to II/R injury and IEC-6 cells subjected to H/R injury. Consequently, FoxM1 expression decreased upon II/R injury, and AS pretreatment significantly reversed the dysregulation of FoxM1 expression in rats subjected to II/R injury (Figures 4A–C). Moreover, we obtained the same results in IEC-6 cells subjected to H/R injury (Figures 4D–F). Our results revealed the protective effect of AS on the II/R-induced dysregulation of intestinal mucosal cell proliferation and barrier function and the regulation of FoxM1 expression.

### 3.5 AS promoted intestinal mucosal cell proliferation and alleviated barrier dysfunction by activating the expression of FoxM1 in rats after II/R injury

**FIGURE 5:**
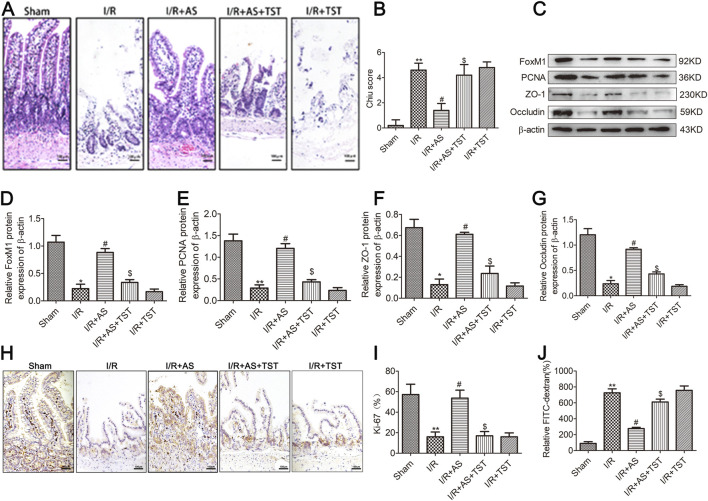
Inhibition of FoxM1 blocks the effects of AS on intestinal epithelial proliferation and barrier function injury in intestinal tissue after II/R injury. **(A,B)** Representative micrographs of intestinal tissues (bar = 100 μm) and Chiu scores of the different groups (n = 5). **(C–G)** Representative Western blot showing FoxM1, PCNA, ZO-1 and occludin protein expression (n = 3). **(H,I)** Immunofluorescence staining for Ki-67 expression in intestinal tissues from different groups (bar = 100 μm) (n = 6). **(J)** Relative levels of FITC-dextran in intestinal tissues from different groups. **P < 0.01 versus sham, ^#^ *P* < 0.05 versus I/R, ^$^ *P* < 0.05 versus I/R + AS.

To further elucidate the mechanism through which AS affects intestinal mucosal cell proliferation and alleviates barrier dysfunction induced by II/R, TST (a FoxM1 inhibitor) was used to inhibit FoxM1 expression in rats after II/R injury. In our study, we first successfully inhibited FoxM1 expression in rats after II/R injury, and proliferation and barrier function injury were evaluated. HE staining revealed that TST significantly reversed the effect of AS pretreatment on II/R injury (Figures 5A,B). The expression level of FoxM1 decreased upon II/R injury, and AS pretreatment significantly reversed the dysregulation of FoxM1 expression and epithelial cell proliferation and barrier dysfunction. Furthermore, TST significantly reversed the upregulation of FoxM1 and intestinal epithelial cell proliferation and barrier function induced by AS pretreatment in rats after II/R injury (Figures 5C–G), and the results of immunofluorescence staining for Ki-67 expression and intestinal permeability assays were the same as the results of Western blotting (Figures 3H–J). Our results reveal that AS ameliorated intestinal epithelial cell proliferation and alleviated barrier dysfunction induced by II/R by activating FoxM1 in rats after II/R injury.

### 3.6 AS promoted cell proliferation and alleviated barrier dysfunction by activating the expression of FoxM1 in IEC-6 cells after H/R injury

**FIGURE 6:**
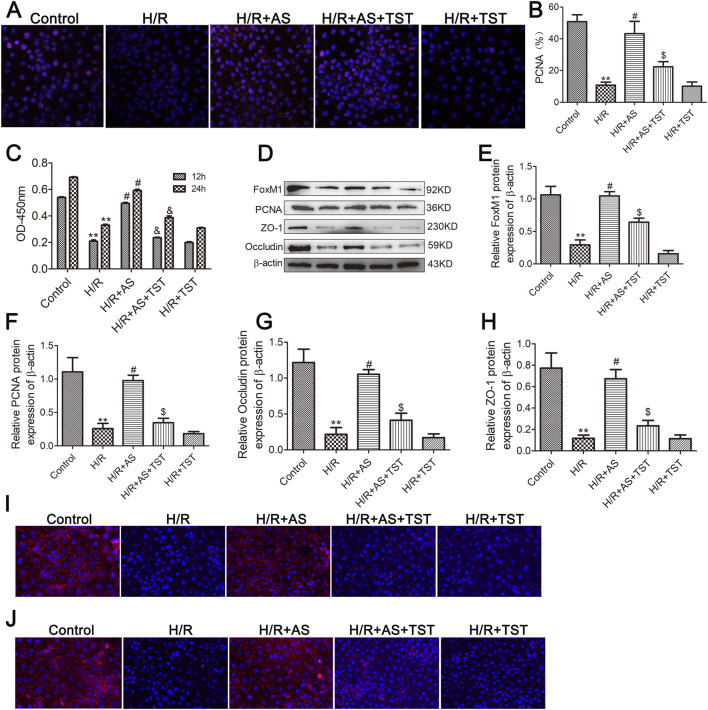
Knockdown of FoxM1 expression blocks the effects of AS on the proliferation and barrier function of IEC-6 cells after H/R injury. **(A,B)** Immunofluorescence staining for PCNA expression in IEC-6 cells (n = 6). **(C)** CCK-8 assay to examine cell proliferation at the indicated time points (n = 6). **(D–H)** Representative Western blot showing FoxM1, PCNA, ZO-1 and occludin protein expression (n = 3). **(I)** Immunofluorescence staining for occludin expression in IEC-6 cells in different groups. **(J)** Immunofluorescence staining for ZO-1 expression in IEC-6 cells in different groups. **P < 0.01 versus control, ^#^ *P* < 0.05 versus H/R, ^$^ *P* < 0.05 versus H/R + AS.

We subsequently employed TST to block FoxM1 expression in IEC-6 cells following H/R injury to corroborate the ability of AS to improve II/R-induced intestinal epithelial proliferation and relieve barrier function injury by activating FoxM1. Immunofluorescence staining revealed that PCNA expression was greater in the AS pretreatment group than in the H/R group and that PCNA expression was lower in the AS pretreatment + TST group than in the AS pretreatment group (Figures 6A–C), and the results of the CCK-8 assay were the same (Figure 6D). Following H/R damage, FoxM1 expression, cell proliferation and barrier function were reduced in IEC-6 cells. WB and immunofluorescence analyses further supported the recovery of the reduced FoxM1 expression, cell proliferation and barrier function following AS pretreatment. TST dramatically reversed the upregulation of FoxM1, cell proliferation and barrier function induced by AS pretreatment in IEC-6 cells (Figures 6E–J). These results verify that AS improved the proliferation of IEC-6 cells caused by H/R and reduced damage to barrier function by activating FoxM1 in IEC-6 cells after H/R injury.

## 4 Discussion

II/R injury is a significant surgical emergency with a high mortality rate and a clinical disorder that poses a threat to life. Severe local and systemic inflammation and multiple organ dysfunction syndrome may ensue from the compromised intestinal barrier dysfunction associated with II/R, increased intestinal permeability, and intestinal flora translocation (Li et al., 2021). After II/R injury, intestinal epithelial proliferation, differentiation and migration are triggered. This restores intestinal barrier function impaired by I/R. Numerous studies have demonstrated the involvement of several signaling pathways in the II/R process (Tang et al., 2025; Zeng et al., 2024). According to earlier research, AS possesses potent antiulcer properties that aid in wound healing. However, the effects of AS on intestinal mucosal cell proliferation and the recovery of barrier function after II/R injury remain unclear. In our study, we first found that 1) AS effectively protects against II/R injury. 2) AS ameliorates II/R injury by promoting intestinal epithelial proliferation and barrier function. 3) AS promotes intestinal epithelial proliferation and barrier function by activating FoxM1 expression.

Mechanical, chemical, immunological and biological barriers are examples of intestinal barriers. The most significant barrier of the intestinal mucosa is the mechanical barrier, which is a full, tight intercellular connection created by intestinal epithelial cells. Tight junction proteins such as occludin and ZO-1 make up the tight intercellular connections of intestinal epithelial cells (Ugalde-Silva et al., 2016). Lumen bacteria, endotoxins, and other poisons can enter the blood and other distant organs when the gut barrier is compromised. Sepsis, multiple organ failure and a high death rate are among the severe clinical outcomes that might result from the damage (Li et al., 2021). In this study, we report significantly decreased intestinal epithelial cell proliferation and expression of occludin and ZO-1 caused by intestinal I/R injury. AS pretreatment promoted intestinal epithelial proliferation and restored the expression of occludin and ZO-1 that was downregulated by II/R injury. The results reveal a protective effect of AS on II/R-induced intestinal barrier function injury.

An important transcription factor, FoxM1 consists of a conserved forkhead domain and a transcription activation domain. These functional domains enable FoxM1 to attach to DNA, triggering the transcription of particular genes and controlling physiological processes in cells. FoxM1 is essential for the development of cancer and metastasis, cell cycle regulation, cell proliferation and DNA repair. According to several studies, FoxM1 plays a role in the regeneration of numerous organs after I/R damage. Zhang et al. reported that forced FoxM1 expression improved the viability and proliferation of H/R-treated H9c2 cells (Zhang et al., 2021). Sinha et al. reported that GSK3β inhibited tubular repair by blocking FoxM1 and that FoxM1 was crucial for renal tubular regeneration after acute kidney damage (Sinha et al., 2020). Our previous studies revealed that FoxM1 promotes the transcription of Nurr1 and intestinal mucosal regeneration after I/R injury (Zu et al., 2019). The inhibition of miR-142 can promote intestinal mucosal cell proliferation and barrier function after I/R injury by directly promoting the expression of FoxM1 (Wang et al., 2025). These findings are consistent with our observations that FoxM1 can promote intestinal regeneration following I/R injury.

Numerous chemical compounds have been shown to activate FoxM1 in various pathogenic diseases. By modifying the deubiquitination of FoxM1, astragaloside IV reduces high glucose-induced trophoblast damage (Li and Zhao, 2025). These findings offer a fresh perspective on the management of gestational diabetes mellitus. Liu et al. reported that plumbagin partially inhibits FoxM1 signaling in glioma cells to demonstrate its anticancer activity (Liu et al., 2015). Our earlier findings revealed that AS activates FoxM1 expression to prevent lung damage caused by II/R (Zheng et al., 2024). In this study, intestinal epithelial proliferation and barrier function were markedly decreased during II/R damage, which may have been facilitated by AS. Hypoxia followed by reoxygenation reduced cell proliferation and barrier function, which is in line with the findings of *in vivo* studies, whereas AS markedly increased cell proliferation and barrier function decreased by H/R injury. Additionally, the ability of AS to enhance cell proliferation and barrier function was reduced when FoxM1 was inhibited by TST. These findings suggest that pretreatment with AS markedly promotes cell proliferation and barrier function induced by II/R injury via activation of FoxM1 expression.

There are various limitations to our investigation. First, we detected the protective effects of AS on II/R injury through the activation of FoxM1 expression; however, the precise mechanisms underlying the link between AS and FoxM1 require further research. Second, our study is based on an animal model, and future research may examine the need for therapeutic applications. Third, the immune responses in the mucosa and submucosa is a very important pathophysiological process and has significant implications of II/R injury (Fukatsu et al., 2006). AS treatment could elicit immune responses in the mucosa and submucosa and the associated alterations particularly those involving mast cells and macrophages (Shestakova et al., 2022). It is of great significance to study the effects of AS treatment on immune responses in the mucosa and submucosa in II/R injury, and this requires further experimental confirmation. In addition, several studies have reported that adverse events and side effects of AS treatment (Kongkaew et al., 2020; Paocharoen, 2010). In our study, we investigated the effects of AS on II/R-induced intestinal epithelial cell proliferation and barrier function in II/R injury. However, we did not detect the adverse events and side effects of AS treatment. Therefore, the adverse events and side effects of AS treatment should be further investigated. In addition, we investigated the protective effects of AS on II/R injury and its mechanistic link to FoxM1 activation. The relationship between AS and FoxM1 regulation remains at a correlative level of “expressional association” without clarification of the specific molecular events. FoxM1 activity validation and upstream or downstream pathway exploration of FoxM1 should be explored in the future study.

## 5 Conclusion

In our study, we investigated the effects of AS on II/R-induced intestinal epithelial cell proliferation and barrier function in rat II/R injury and IEC-6 cell H/R injury models. Our results show that AS alleviated II/R-induced intestinal morphological damage and promoted intestinal epithelial cell proliferation and recovery from barrier dysfunction via activation of the expression of FoxM1. Our results suggest the potential use of AS as a novel candidate to alleviate II/R injury.

## Data Availability

The original contributions presented in the study are included in the article/supplementary material, further inquiries can be directed to the corresponding author.
